# The use of a modified Delphi technique to develop a critical appraisal tool for clinical pharmacokinetic studies

**DOI:** 10.1007/s11096-022-01390-y

**Published:** 2022-03-20

**Authors:** Alaa Bahaa Eldeen Soliman, Shane Ashley Pawluk, Kyle John Wilby, Ousama Rachid

**Affiliations:** 1 College of Pharmacy, QU Health, Qatar University, Doha, Qatar; 2 Children’s & Women’s Health Centre of British Columbia, Provincial Health Services Authority, British Columbia, Canada; 3 Faculty of Pharmaceutical Sciences, University of British Columbia, British Columbia, Canada; 4 College of Pharmacy, Faculty of Health, Dalhousie University, Halifax, Nova Scotia Canada; 5 Biomedical and Pharmaceutical Research Unit, QU Health, Qatar University, Doha, Qatar; 6 Correspondence: College of Pharmacy, QU Health, Qatar University, P.O. Box 2713, Doha, Qatar

**Keywords:** Critical appraisal, Critical appraisal tool, Clinical pharmacokinetics, Pharmacokinetics, Quality markers, Reporting checklist

## Abstract

**Background:**

Critical appraisal aids in assessing the quality of scientific literature, which is central to the practice of evidence-based medicine. Several tools and guidelines are available for critiquing and assessing the quality of specific study types. However, limited guidance exists for critical appraisal of clinical pharmacokinetic studies.

**Aim:**

We aimed to achieve experts’ consensus regarding the quality markers for clinical pharmacokinetic studies in an attempt to develop a critical appraisal tool.

**Method:**

Quality markers related to clinical pharmacokinetic studies, were derived from the published literature and categorized according to manuscript reporting domains (abstract, introduction/background, methodology, results, discussion, and conclusion). Questions that aid in appraising pharmacokinetic studies were formulated from these quality markers. Experts were involved in a modified Delphi process to achieve a consensus regarding the formulated questions. The proposed tool was pilot tested on 30 recently published clinical pharmacokinetic studies. Inter-observer agreement was measured to determine the reliability of the included items.

**Results:**

Twenty-five experts consented to participate. Three rounds of a modified Delphi survey were required to generate a consensus for a 21-item tool aimed at appraising the quality of clinical pharmacokinetic studies. When applied to 30 recently published clinical pharmacokinetic studies, most items scored fair to moderate levels of agreement (61.90–95.24%).

**Conclusion:**

The clinical pharmacokinetic critical appraisal tool (CACPK) developed in this study consisted of 21 items aimed at helping an end-user to determine the quality of a pharmacokinetic study. Further studies are warranted to reaffirm the validity and reliability of the CACPK tool.

**Supplementary Information:**

The online version contains supplementary material available at 10.1007/s11096-022-01390-y.

## Impact Statements

- Specific dimensions of quality related to clinical pharmacokinetic research include study design, conduct, analysis, results validity, clinical relevance, and quality of reporting.
- Developing a critical appraisal tool to evaluate clinical pharmacokinetic studies will help end-users to apply evidence-based medicine to clinical practice and future research.
- The clinical pharmacokinetic critical appraisal tool (CACPK) developed in this study consisted of 21 items aimed at helping end-users to appropriately assess the quality of pharmacokinetic studies.
- The currently developed CACPK tool shall undergo periodic improvement in order to keep pace with changes in the literature and to maintain the practical benefits to the field of clinical pharmacokinetics.

## Introduction

The practice of evidence-based medicine (EBM) integrates individual clinician’s expertise and experience with the best available clinical evidence from empirical research [1, 2]. Central to the practice of EBM is the critical appraisal of published literature, in which the relevance, quality, and the trustworthiness of a published study is systematically assessed [2]. The critical appraisal process helps in assessing the validity, reliability, and quality of the published scientific knowledge. Therefore, several generic and specific critical appraisal tools for varying study designs were developed. Design-specific critical appraisal tools contain items that critique the methodological quality of the study design [3]. Generic critical appraisal tools help in appraising quantitative and qualitative studies in general [4]. Accordingly, the critical appraisal process aids in assessing the quality of the study results, and how they are interpreted and applied in health policymaking, therapeutic decision, and clinical practice [5]. Therefore, selecting the most appropriate critical appraisal tool is essential for the application of evidence-based practice [6].

The application of pharmacokinetic principles in clinical settings is considered an integral part of pharmaceutical care provision by the pharmacist [7]. Clinicians aim to improve a patient’s response to the drug dosage regimen and to minimize toxicity by designing an individualized dosage regimen through the application of clinical pharmacokinetic concepts [8]. This process is aided by literature review of available evidence extracted from clinical pharmacokinetic studies, which have to meet high standards of quality to ensure the delivery of safe and effective therapeutic regimen [9].

There are many developed and published critical appraisal tools and reporting quality checklists to guide researchers to assess the quality of the published research. Reporting checklists, however, are not synonymous to critical appraisal tools. A study may meet the required aspects for reporting, but may fail to meet the expected quality standards. Furthermore, some reporting checklists may not assess important dimensions of quality related to the study design, conduct, analysis, clinical relevance, and result validity [9]. Additionally, currently published critical appraisal tools are not highly specific to determine the methodological quality and validity of clinical pharmacokinetic studies. Most of the available tools lack specific items that help in analyzing the published articles in depth [10].

To our knowledge, two studies were conducted to assess the quality of reporting clinical pharmacokinetic studies [11, 12]. The first study was a systematic review, which evaluated the quality of reporting of pharmacokinetic studies of antibiotics in patients with sepsis receiving continuous renal replacement therapy [11]. In this systematic review, the researchers found that none of the identified articles during their systematic search reported the full set of parameters that help end-users interpret the reported results. Furthermore, 20% of the published pharmacokinetic trials did not contain the fundamental pharmacokinetic parameters [11]. Consequently, reporting guidelines for clinical pharmacokinetic studies (The ClinPK Statement) were issued to guide investigators in reporting clinical pharmacokinetic studies. The developers designed a yes/no checklist that was composed of 24 items to guide researchers in reporting the minimum required information in clinical pharmacokinetic studies [12]. While offering a valuable guideline for reporting the findings of clinical pharmacokinetic studies, The ClinPK Statement has its drawbacks and did not cover some important dimensions of quality. Another tool, Grading and Assessment of Pharmacokinetic-Pharmacodynamic Studies (GAPPS), was recently published to assess the strength of evidence extracted from pediatric PKPD antibiotic studies [13].

A critical appraisal tool with broad application that aids clinicians in appraising and assessing the quality of published clinical pharmacokinetic studies does not exist. Developing an understanding of quality markers for clinical pharmacokinetic studies that clinicians consider important would aid in the creation of a tool aimed at assessing the quality of published clinical pharmacokinetic studies.

### Aim

We aimed to achieve an expert consensus regarding the quality markers of clinical pharmacokinetic studies with an attempt to develop a critical appraisal tool through a modified Delphi technique.

### Ethics approval

This study was approved by the Qatar University Institutional Review Board: QU-IRB 970-E/18. Data were analyzed using SPSS version 24 (IBM SPSS® Statistics for Windows, version 24; IBM Corp, Armonk, NY, USA).

## Method

### Use of pre-selected set of preliminary quality markers

A pre-selected set of preliminary quality markers of clinical pharmacokinetic studies identified systematically [14] was used to build a questionnaire, comprising potential identified candidate items into a design-specific critical appraisal tool. These items were categorized into relevant study sections, including title, abstract, background, methods, results, discussion, conclusion and other.

### Sampling and selection of Delphi experts

A purposive sampling method was used to select participants who met the study’s eligibility criteria. Potential participants were approached as expert panelists if they were considered as any of the following:

- academic experts with a designation that reflects their direct involvement in clinical pharmacokinetics as evidenced in their research and teaching portfolios.
- clinicians who had experience in the application of clinical pharmacokinetic principles or the provision of therapeutic drug monitoring in their clinical practice. These individuals should have experience in interpreting the findings of clinical pharmacokinetic studies and applying these to their patients.
- pharmaceutical industry researchers with years of experience in relevant positions involving clinical pharmacokinetic research.
- individuals in regulatory bodies who assess clinical pharmacokinetic studies when making decisions for their respective health authorities, e.g. FDA.

Potential participants were requested to state their area of expertise related to clinical pharmacokinetics, number of years in the field of clinical pharmacokinetics, current geographical location of practice and a global self-assessed competence in the field of clinical pharmacokinetics (none, little, average, advanced).

### Modified Delphi survey

A modified Delphi process was utilized via an online survey platform (SurveyMonkey®) in order to build consensus amongst experts regarding each item considered for inclusion in the tool. An inventory of quality markers to evaluate pharmacokinetic studies [14] was used to identify potential items through a systematic approach. These items were revised scientifically and linguistically, reworded, and reduced after several iterations with the research team members prior to being sent to the study participants. A large number of participants could be included in the rounds through using modified Delphi design because of the lack of geographical restrictions. Participants could express their opinions freely, as their answers were anonymous. Therefore, a dominance that might occur during face-to-face interactions and any bias introduced by moderators were avoided. Panelists in this study were recruited from different countries and practice settings like academia, industry, clinical practice, and regulatory authorities. Thus, each participant could share their perspective in the field of clinical pharmacokinetics and help in generating new ideas that helped in broadening the knowledge base of other participants.

### Modified Delphi process

Round 1 of this modified Delphi process utilized a questionnaire comprising potential identified candidate items. Experts were presented with individual candidate items and were requested to state their level of agreement that the item should be included using a 5-point Likert scale (1 = strongly disagree, 2 = disagree, 3 = neither agree nor disagree, 4 = agree, 5 = strongly agree). For each candidate item, participants were given the option to provide free-text comments to support their rating or to suggest changes to the item. Upon completion of the potential items generation, participants were given the option to nominate additional items for consideration.

Consensus was set *a priori* at 75% agreement with any one of the available outcomes (inclusion, exclusion, modification). Inclusion was met if 75% or more of the participants rated an item as agree or strongly agree. Exclusion was met if 75% or more of participants rated an item as disagree or strongly disagree. Item modification was considered if neither inclusion nor exclusion were met after which the item was modified based on participants’ feedback. If after a modification, an item still did not meet the requirements for inclusion or exclusion and the percentage change in consensus was less than 15%, it was excluded from the final tool to be used for critical appraisal of clinical pharmacokinetic studies (CACPK). For each additional Delphi round following round 1, participants were given the blinded aggregate comments from all other participants for each item. As the aim of the study was to create a tool to be used to assess the quality of clinical pharmacokinetic studies, the researchers sought to gauge the participants’ opinion of an effective means of rating each item included in the tool. Consequently, an additional section of the survey asked participants to rate various scales of measurement that could be used to assess each item in the finalized tool. These initially included options of “yes/no” and a 4-point scale ranging from “poor, fair, good, to excellent”. Authors were also open to additional options presented during the modified Delphi. The experts were also provided a free-text box to suggest additional rating systems. Participants were given four weeks to complete each Delphi round with reminder emails being sent after three weeks. All participants’ responses were kept anonymous from the other participants.

### Pilot testing

The CACPK final tool was applied to 30 clinical pharmacokinetic studies in order to test validity and feasibility of the tool in evaluating published literature. Ten studies were purposively selected each from three journals that focus on clinical pharmacokinetics (Clinical Pharmacokinetics, International Journal of Pharmacokinetics, Journal of Pharmacokinetics and Pharmacodynamics). Articles included randomized clinical pharmacokinetic trials, evaluations of drug interactions, population pharmacokinetic studies, and bioequivalence studies. Studies including less than five participants were excluded from the pilot testing. Application of the quality tool was performed by four end-users (AS, SP, OR, KW) in duplicate. Included articles were divided equally at random amongst raters so that each rater evaluated 15 articles and each article was evaluated twice. Raters were also asked to record the time to complete the quality assessment of each article evaluated. Outcomes of ‘No, I don’t know, and Not applicable’ were grouped together. Cohen’s Kappa was used to assess level of agreement between raters [15]. Kappa values of less than 0 were rated as less than the chance of agreement; 0.01–0.20, slight agreement; 0.21–0.40, fair agreement; 0.41–0.60, moderate agreement; 0.61–0.80, substantial agreement; 0.81–0.99, almost perfect agreement. Average time to complete each assessment was assessed for feasibility of the tool.

## Results

### Participants

One hundred and nineteen potential expert panelists, identified by the research team or through snowballing, were invited to take part in this study. Personal invitations were sent via email. Twenty-five panelists agreed to participate in the modified Delphi process. The sociodemographic characteristics of the panelists of the modified Delphi are shown in Table 1.

**Table 1:** Sociodemographic characteristics of modified Delphi panelists

Variables	Percentage (Actual number)
*Filed of experience*	
Clinicians	56% (14/25)
Academic sector	20% (5/25)
Industrial sector	16% (4/25)
Regulatory sector	4% (1/25)
Project director	4% (1/25)
*Grographical distribution*	
Canada	52% (13/25)
USA	24% (6/25)
Qatar	24% (6/25)

The average level of experience of the participants in the field of clinical pharmacokinetics was 8.5 years with 56% self-identified as clinicians with relevant practice experience in clinical pharmacokinetics. Around 96% of the participants rated themselves with an average or advanced level of competence in clinical pharmacokinetics.

### Consensus through modified Delphi rounds

Three rounds of the modified Delphi were conducted. Response rates for each round were 24/25 (96%), 23/25 (92%), and 15/25 (60%), respectively.

**Fig. 1:**
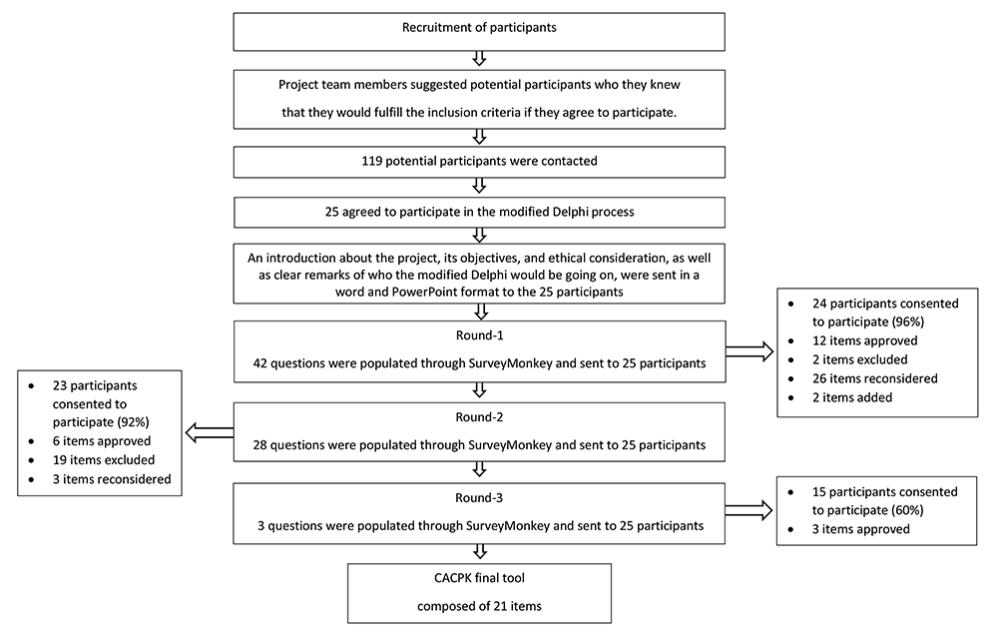
The modified Delphi flow chart of all rounds

Forty items were identified through systematic search of the literature and were included in the first round of the Delphi. Two additional control items that were not deemed relevant to clinical pharmacokinetics were included to gauge the integrity of the participant responses for a total of 42 items (Supplement 1). At the end of round 1, the panel reached consensus on inclusion of 12 items (28.6%). These items were included in the CACPK final tool and were not entered into round 2. Two items met consensus for exclusion (4.8%). Both control items met consensus for exclusion and were not included in any additional rounds of the Delphi. Furthermore, two additional items were added based on participant suggestions/comments. The remaining 26 items were amended based on participants’ comments and were re-entered into round 2 (Fig. 1).

In round 2, 28 items were presented to the participants for consideration. At the end of the round, 6 items (21.4%) reached consensus for inclusion in the CACPK final tool, while 19 items (67.9%) reached consensus for exclusion. The remaining three items (10.7%) were reformulated based on participant’s feedback and were included in round 3.

Round 3 comprised of 3 items in which all (100%) reached consensus for inclusion into the CACPK final tool. Consensus rates for each of the included item along with the round of the modified Delphi in which they were approved are summarized in Table 2. Participant consensus regarding the choice of rating of each item in the CACPK final tool was for items to be rated as ‘Yes, No, I don’t know, and Not applicable’.

**Table 2:** Consensus rate of the items included in the final tool for Critical Appraisal of Clinical Pharmacokinetic (CACPK) studies

Item		Consensus [N (%)]^*^	Round of consensus^**^
Background			
1	Was a clear description of the objectives of the study provided?	21/21 (100)	1
2	Was a valid and comprehensive rationale provided to support the purpose of the study?	13/15 (86.7)	3
Design			
3	Was the chosen study design appropriately selected and justified?	20/21 (95.2)	1
4	Was the dosing (dose, route of administration, dosing interval) of the drug in the study justified for the intended study?	19/21 (90.5)	1
5	Were the endpoints of the study appropriate to answer the objectives of the study?	19/21 (90.5)	1
6	Were the exclusion criteria of participants included AND appropriate for the intended outcomes of the study?	18/21 (85.7)	1
7	Where applicable, were the relevant baseline characteristics of the participants adequately described?	14/15 (93.3)	3
8	Were plausible interacting covariates described a priori or in post hoc evaluation?	18/19 (94.7)	2
9	Was the description of the used sample analysis methods or citations of prior validation studies provided in the publication or affiliated appendix?	16/19 (84.2)	2
Sampling			
10	Was the method of data sampling appropriate for the study?	19/21 (90.5)	1
11	Was a clear description of the sampling site and the sampling interval (the exact times at which samples are obtained) provided and justified?	19/21 (90.5)	1
12	Was the number of half-lives elapsed within the sampling period appropriate for the analyzed drug?	17/19 (89.5)	2
13	Were sample storage conditions appropriate and described in a manner that could be accurately replicated?	20/21 (95.2)	1
14	If applicable, was there a clear description of the pharmacokinetic model, its development, validation and justification for use?	20/21 (95.2)	1
15	Was the described population pharmacokinetic approach validation method appropriate for the analysis?	15/15 (100)	3
16	Were the essential pharmacokinetic parameters required to make the results applicable in clinical settings addressed?	16/19 (84.2)	2
17	Were the pharmacokinetic equations used to calculate patient pharmacokinetic parameters disclosed or cited within the article?	16/19 (84.2)	2
Applied Statistics			
18	Were the chosen statistical tests and software to perform the statistical analysis appropriate to achieve the study objectives?	19/21 (90.5)	1
Results			
19	Were all patients enrolled in the study accounted for?	17/19 (89.5)	2
20	In the event of missing data or outliers, was the process for analysis justified and appropriate?	19/21 (90.5)	1
21	Were appropriate summary statistics to describe centrality and variance used to document the pharmacokinetic results?	19/21 (90.5)	1

^*^The denominator varies for each item and reflects to the total number of responses for each item in the round in which it was included. ^**^Represents the round of the modified Delphi for which the item was included

The CACPK final tool included 21 items that met consensus for inclusion (Table 3). The tool was divided into five sections: background, design, sampling, applied statistics, and results. The final rating scale that was used to help the end-users to assess the quality of published clinical pharmacokinetic studies was “Yes, No, I Do Not Know, and Not Applicable”.

**Table 3:** Final tool for Critical Appraisal of Clinical Pharmacokinetic studies (CACPK)

Critical Appraisal of Clinical Pharmacokinetic Studies (CACPK) Tool	
**Appraising Background**	
1. Was a clear description of the objectives of the study provided? • Authors should provide a clear statement of the objectives of the research to clarify the purpose and the scope of the study.	Yes No I Do Not Know Not Applicable Comments: _____________________________
2. Was a clear and comprehensive rationale provided to support the purpose of the study?	Yes No I Do Not Know Not Applicable Comments: ______________________________
**Appraising Study Design and Experimental Methods**	
3. Was the chosen study design appropriately selected and justified?	Yes No I Do Not Know Not Applicable Comments: ______________________________
4. Was the dosing (i.e. dose, route of administration, and dosing interval) of the drug in the study justified for the intended study? **Examples**: • Authors should justify the use of single-dose versus steady-state analysis.	Yes No I Do Not Know Not Applicable Comments: ______________________________
5. Were the outcome measures endpoints of the study appropriate to address the objectives of the study?	Yes No I Do Not Know Not Applicable Comments: ______________________________
6. Were the exclusion criteria of participants included AND appropriate for the intended outcomes of the study? • The exclusion criteria should be relevant to assist with decreasing significant confounders (e.g. co-administration of drugs, organ impairment, and special populations) that may impact outcomes.	Yes No I Do Not Know Not Applicable Comments: ______________________________
7. Where applicable, were the relevant baseline characteristics of the participants adequately described? **Examples**: • Sex, race, age, weight, height, concomitant disease, administered medications, smoking status, pregnancy, severity of illness that may affect pharmacokinetic parameters, renal function, and hepatic function. **Note**: Please refer to Appendix-1 Patient Demographics (Supplement 4) for further clarification.	Yes No I Do Not Know Not Applicable Comments: ______________________________
8. Were plausible interacting covariates described *a priori* or in post hoc evaluation? **Examples**: • Demographic variables, laboratory values, concomitant medications, and relevant disease states to the drug being studied.	Yes No I Do Not Know Not Applicable Comments: ______________________________
9. Was the description of the used biological sample analytical methods sample analysis methods or citations of prior validation studies provided in the publication or affiliated appendix? **Examples**: • Chromatography type. • Detection type. • Assay characteristics: mobile phase composition, gradient and flow rate, chromatographic column (packing material, dimensions). • Analytical runtime. • Operating temperature. • Detection parameters. • Validation method: specificity, recovery, linearity and sensitivity, the stability of the assay and its reproducibility. Refer also to EMA/FDA guidelines for bioanalytical method validation.	Yes No I Do Not Know Not Applicable Comments: ______________________________
10. Was the method of data sampling of analytics appropriate for the study? **Examples**: • First vs. second order absorption, and lag time. • Evaluating for nonlinearity requires multiple dose levels and a complete profile is recommended. • Researchers obtain these data from previously conducted studies with completed concentration-time profile (e.g. phase I studies). • The method of data sampling should reference previously validated quantitative bioanalytical methods and if those are not available then the full description or defense of data sampling should be included.	Yes No I Do Not Know Not Applicable Comments: ______________________________
11. Was a clear description of the sampling site provided and justified? **Examples**: • Sampling site should be consistent for all subjects in the study. • Arterial sampling is preferable during frequent sampling schedule. • Arterial sampling is more representative of the delivered concentration to the effect site in the case of peripheral elimination. • Arterial sampling is preferable when administering a drug that has a short duration of action or fast onset of action.	Yes No I Do Not Know Not Applicable Comments: ______________________________
12. Was the number of half-lives elapsed within the sampling period appropriate for the analyzed drug? **Examples**: • Sampling interval should not exceed the expected half-life of the studied exponential phase (fast distribution, slow distribution and elimination).	Yes No I Do Not Know Not Applicable Comments: ______________________________
13. Were sample storage conditions appropriate and described in a manner that could be accurately replicated? **Examples**: • Sample storage, temperature, use and description of anticoagulants, stabilizers, centrifugation etc.	Yes No I Do Not Know Not Applicable Comments: ______________________________
14. If applicable, was there a clear description of the pharmacokinetic model, its development, validation and justification for use? It is recommended to provide the following details about the selected modeling process: • Description of studies from which dataset was driven • Model structure • Validated software for the pharmacokinetic analysis • Criteria for accepting valid model’s parameters • Fitting procedure defined prior to the initiation of the analysis. • A reasonable assumption based on which the scheme for weighting is considered to be appropriate and the transformation of data [e.g. logarithmic transformation to achieve the homoscedastic (constant) variance requirements] should be provided.	Yes No I Do Not Know Not Applicable Comments: ______________________________
15. Was the described population pharmacokinetic approach validation method appropriate for the analysis? 1- Basic internal method 2- Advanced internal method 3- External model evaluation **Note**: Please refer to Appendix-2 Model Evaluation (Supplement 5) for further clarification.	Yes No I Do Not Know Not Applicable Comments: ______________________________
16. Were the essential pharmacokinetic parameters required to make the results applicable in clinical settings included? **Examples**: • Total clearance (CL), Hepatic clearance, Renal clearance, Volume of distribution at steady state (Vss), Blood/plasma concentration ratio, Terminal half-life (t_1/2_ Z), Fraction of the unbound drug in plasma (fu), Absorption rate constant (Ka),C_min_, C_max_, t_max_,, AUC, etc.	Yes No I Do Not Know Not Applicable Comments: ______________________________
17. Were the pharmacokinetic equations used to calculate the patient’s pharmacokinetic parameters presented or cited within the article? **Examples**: • Equations used to calculate the following pharmacokinetic parameters: creatinine clearance, body weight calculations, Michaelis Menten, volume of distribution	Yes No I Do Not Know Not Applicable Comments: ______________________________
**Appraising Applied Statistics**	
18. Were the chosen statistical tests and software to perform the statistical analysis appropriate to achieve the study objectives?	Yes No I Do Not Know Not Applicable Comments: ______________________________
**Appraising Results**	
19. Were all patients enrolled in the study accounted for? **Examples**: • Description of patient screening, enrollment, run-in or wash out phases, study period and follow-up periods are adequately described. Any loss to follow-up or withdrawals are described.	Yes No I Do Not Know Not Applicable Comments: ______________________________
20. In the event of missing data or outliers, was the process for analysis justified and appropriate?	Yes No I Do Not Know Not Applicable Comments: ______________________________
21. Were appropriate summary statistics to describe centrality and variance used to present the pharmacokinetic results? **Examples**: • Descriptive statistics such as confidence interval, standard deviation, mean, median, range, interquartile range, standard error and trimmed range	Yes No I Do Not Know Not Applicable Comments: ______________________________

### Pilot testing

Thirty recently published articles focused on clinical pharmacokinetics were assessed for quality (Supplement 2) using the quality assessment tool developed through the modified Delphi (CACPK final tool). A majority of items (12/21) were scored at fair to moderate agreement. Of note, items 13 (were sample storage conditions appropriate and described in a manner that could be accurately replicated?), 16 (were the essential pharmacokinetic parameters required to make the results applicable in clinical settings addressed?), 18 (were the chosen statistical tests and software to perform the statistical analysis appropriate to achieve the study objectives?) and 21 (were appropriate summary statistics to describe centrality and variance used to document the pharmacokinetic results?) scored substantial to almost perfect agreement. Kappa values could not be calculated for items 2 and 5 as all respondents answered ‘yes’ with 100% agreement. Items 3 and 10 scored less than the chance of agreement.

Applying this CACPK final tool to clinical pharmacokinetic studies took an average of 28.5 min (range 14–90 min). No raters expressed any difficulty in understanding any of the items or applying these to the studies.

## Discussion

### Statement of key findings

The CACPK tool (Supplement 3) developed in this study offers new understanding of quality markers that should be utilized for appraising published clinical pharmacokinetic studies via pharmacokinetics experts consensus. Together with the CACPK tool, two additional appendices (Supplements 4 and 5) were created in case further clarification is needed about items 7 and 15 in the tool, respectively. This tool with its two appendices guide researchers through answering 21 questions to determine the quality of published clinical pharmacokinetic studies. The tool was developed in a format similar to other critical appraisal tools like a measurement tool to assess systematic reviews (AMSTAR) to facilitate the appraising process and was composed of four sections: appraising background, appraising study design and experimental methods, appraising applied statistics, and appraising results [16].

### Strengths and weaknesses

The development of a critical appraisal tool for clinical pharmacokinetic studies adds much value to the existing literature as it complements currently available checklists by assessing dimensions of quality of clinical pharmacokinetic studies. This tool, with further testing of reliability and validity, can be potentially used to guide clinicians and policymakers to evaluate the quality of published articles to take clinical decisions and develop policies by applying EBM. Furthermore, stakeholders who work in the academic sector can use it to teach students how to appraise this type of study.

Despite these strengths, we need to acknowledge the limitations of this study. Participants’ identification was anonymous as researchers, which prevented the ability to correlate responses to professional experience. Furthermore, we received a declining response rate with each round of the survey leading to overemphasized response from a smaller number of participants in the latter rounds of the study.

### Interpretation

The aim of developing the CACPK tool was to assess the quality of studies, which has several dimensions including study design, conduct, analysis, clinical relevance, results validity and quality of reporting. While other clinical pharmacokinetic guidelines have focused on assessing the quality of reporting of clinical pharmacokinetic research, these tools do not directly address whether the methods used were of high quality. This tool thus assesses the appropriateness of the execution of methods used in carrying out the desired clinical pharmacokinetic analysis.

Pilot testing of the clinical pharmacokinetic critical appraisal tool amongst four individuals resulted in a majority (12/21) of items on the tool scoring fair to moderate agreement. While these four individuals did have considerable experience in the application of clinical pharmacokinetics, this result may not be replicated if clinicians with little to no understanding of clinical pharmacokinetic principles use this tool. Application of this tool in assessing the quality of a clinical pharmacokinetic study did however appear to be reasonably feasible in regards to time requirement with the average study taking under 30 minutes to appraise.

To our knowledge, this study is among the few ones to identify quality markers of clinical pharmacokinetic studies for developing a specific critical appraisal tool with broad application. One of the main strengths of this study was the robustness of the methodology used to develop clinical pharmacokinetics critical appraisal tool. In the modified Delphi process, we were keen to recruit experts who represent all clinical pharmacokinetic stakeholders (clinicians, researchers, individuals in academic and industrial sectors and policymakers). The inclusion of different stakeholders allowed us to enrich our tool with different perspectives from different end-users.

### Further research

Further research can be conducted to further assess the validity, reliability, and overall utility of the tool. Research should also be conducted to determine how the presence or absence of most important markers of quality for clinical pharmacokinetic studies affects study results and interpretation. We acknowledge that the field of clinical pharmacokinetics is vast and that end-users of clinical pharmacokinetic research extends to a vast array of clinicians. It is therefore unlikely that we included expert panelists from all areas relevant to clinical pharmacokinetics.

## Conclusion

This study aimed to achieve expert consensus regarding pre-identified set of quality markers for the appraisal of clinical pharmacokinetic studies. Through this modified Delphi process, a list of questions gauging the overall quality of clinical pharmacokinetic studies was developed that allowed for critical appraisal. Work presented in this study provides a critical appraisal tool (CACPK) with broad application to clinical pharmacokinetic studies.

## Electronic Supplementary Material

Below is the link to the electronic supplementary material.

Supplementary Material 1

Supplementary Material 2

Supplementary Material 3

Supplementary Material 4

Supplementary Material 5

## Data Availability

Not applicable.
