# Supplementary material for: The use of a modified Delphi technique to develop a critical appraisal tool for clinical pharmacokinetic studies
Source: Int J Clin Pharm. 2022 Mar 20;44(4):894–903. doi: 10.1007/s11096-022-01390-y (PMC9393138; doi:10.1007/s11096-022-01390-y)
Supplement: Supplementary file 1 — Supplementary Material 1 [file 11096_2022_1390_MOESM1_ESM.docx]

| **Supplement 1:** Questions disclosed to round-1 | |
| --- | --- |
| **Questions** | **5 Points Likert-scale** |
| Q1) Delphi Study Consent Form | 1. Agree 2. Disagree |
| Q2) Did the title concisely reflect the topic discussed in the paper?   - The title should reflect the name of the analyzed medication, and comparator (if applicable), the targeted patient population, and the study design. | \| 1 \| 2 \| 3 \| 4 \| 5 \| \| --- \| --- \| --- \| --- \| --- \| |
| Q3) Was an adequate summary of the article provided to the readers within the abstract?   - A brief description of the knowledge gap, the objectives, summary of the methods, the results of the primary objectives and the main conclusion should be provided. | \| 1 \| 2 \| 3 \| 4 \| 5 \| \| --- \| --- \| --- \| --- \| --- \| |

| **Questions** | **5 Points Likert-scale** |
| --- | --- |
| Q4) Was a comprehensive introduction provided that explained the rationale behind the conduction of the study?  Authors may provide information related to:   - Stages of the analyzed drug development. - Known aspects of the drug's absorption, distribution, metabolism and elimination. - Previous studies. - What will be added to the existing body of knowledge with their proposed study. | \| 1 \| 2 \| 3 \| 4 \| 5 \| \| --- \| --- \| --- \| --- \| --- \| |
| Q5) **Was a clear description of the objectives of the study provided?** Authors should provide a clear statement of the objectives of the research to clarify the purpose and the scope of the study. | \| 1 \| 2 \| 3 \| 4 \| 5 \| \| --- \| --- \| --- \| --- \| --- \| |
| Q6) Was the chosen study design appropriately selected and justified?  Example:   - Immediate release formulation, single dose study design is often recommended. - Sustained release formulation and medication with a long half-life or high intra-patient variability, parallel study design or steady-state design are often recommended. - Bioequivalence studies, crossover design is often recommended with a washout period between the administered interventions. | \| 1 \| 2 \| 3 \| 4 \| 5 \| \| --- \| --- \| --- \| --- \| --- \| |
| Q7) Was the dosing of the drug in the study justified and/or referenced for the intended study? | \| 1 \| 2 \| 3 \| 4 \| 5 \| \| --- \| --- \| --- \| --- \| --- \| |
|  |  |
| Q8) Were the endpoints of the study clearly relevant for the intended use of the drug?   - The endpoints should be directly related to the objectives. | \| 1 \| 2 \| 3 \| 4 \| 5 \| \| --- \| --- \| --- \| --- \| --- \| |
| Q9) Did the eligibility criteria of participant inclusion reflect the population of interest for which the drug is intended for use?   - The inclusion criteria should allow authors to choose study participants appropriately who are representative of the targeted population to answer the main study question. | \| 1 \| 2 \| 3 \| 4 \| 5 \| \| --- \| --- \| --- \| --- \| --- \| |
| Q10) Were the exclusion criteria of participants appropriate for the intended outcomes of the study?   - Exclusion criteria should not be restricted to an extent that interferes with the generalizability of the study results. | \| 1 \| 2 \| 3 \| 4 \| 5 \| \| --- \| --- \| --- \| --- \| --- \| |

| **Questions** | **5 Points Likert-scale** |
| --- | --- |
| Q11) Was the study setting/location relevant to where the drug would be used?   - Authors should reflect on the setting and/or location of the study. | \| 1 \| 2 \| 3 \| 4 \| 5 \| \| --- \| --- \| --- \| --- \| --- \| |
| Q12) Was the blinding of the study participants, the investigators and/or those analyzing the data appropriate while the study was being conducted? | \| 1 \| 2 \| 3 \| 4 \| 5 \| \| --- \| --- \| --- \| --- \| --- \| |
| Q13) Was the method of data sampling appropriate for the study?  Example:   - Extensive data sampling is essential to select the most appropriate structural model number of compartments, first vs second order absorption, and lag time. - To determine the linearity of pharmacokinetics, sparse data sampling is recommended. Researchers obtain these data from previously conducted studies with completed concentration-time profile e.g. phase I studies. | \| 1 \| 2 \| 3 \| 4 \| 5 \| \| --- \| --- \| --- \| --- \| --- \| |
| Q14) Was a clear description of the sampling site and the sampling interval (the exact times at which samples are obtained) provided and justified?  Example:   - Arterial sampling is preferable during frequent sampling schedule. - Arterial sampling is more representative of the delivered concentration to the effect site in the case of peripheral elimination. - Arterial sampling is preferable when administering a drug that has a short duration of action or fast onset of action. - Sampling interval should not exceed the expected half-life of the studied exponential phase (fast distribution, slow distribution and elimination) | \| 1 \| 2 \| 3 \| 4 \| 5 \| \| --- \| --- \| --- \| --- \| --- \| |
| Q15) Accurate participant follow-up was clearly described and rationalized?  Example:   - Monitoring parameters (e.g. signs and symptoms of disease or side effects of the given medication, lab data, etc.) to be collected in each period should be documented. | \| 1 \| 2 \| 3 \| 4 \| 5 \| \| --- \| --- \| --- \| --- \| --- \| |
|  |  |
|  |  |

| **Questions** | **5 Points Likert-scale** |
| --- | --- |
| Q16) Were sample storage conditions described in a manner that could be accurately replicated?  Example, use of:   - Anticoagulants - Stabilizers - Centrifugation - Temperature | \| 1 \| 2 \| 3 \| 4 \| 5 \| \| --- \| --- \| --- \| --- \| --- \| |
| Q17) Was there a clear description of the pharmacokinetics model, its development, and justification for use?   - It is recommended to provide the following details about the selected modeling process: - Description of studies from which dataset was driven - Model structure - Validated software for the pharmacokinetics analysis - Criteria for accepting valid model’s parameters - Fitting procedure defined prior to the initiation of the analysis. A reasonable assumption based on which the scheme for weighting is considered to be appropriate and the transformation of data (e.g. logarithmic transformation to achieve the homoscedastic (constant) variance requirements) should be provided. | \| 1 \| 2 \| 3 \| 4 \| 5 \| \| --- \| --- \| --- \| --- \| --- \| |
| Q18) Were plausible interacting covariates (demographic variables, laboratory values, co-medication, environmental factors and disease states) described a priori? | \| 1 \| 2 \| 3 \| 4 \| 5 \| \| --- \| --- \| --- \| --- \| --- \| |
| Q19) Was the description of the used apparatus for analysis adequate?  Example:   - Chromatography type. - Detection type. - Assay characteristics: mobile phase composition, gradient and flow rate, chromatographic column (packing material, dimensions). - Analytical runtime. - Operating temperature and detection parameters. - Validation method: specificity, recovery, linearity and sensitivity, the stability of the assay and its reproducibility. | \| 1 \| 2 \| 3 \| 4 \| 5 \| \| --- \| --- \| --- \| --- \| --- \| |
|  |  |
|  |  |

| **Questions** | **5 Points Likert-scale** |
| --- | --- |
| Q20) Were the pharmacokinetics equations used to calculate different patient parameters (e.g. creatinine clearance) disclosed within the article? | \| 1 \| 2 \| 3 \| 4 \| 5 \| \| --- \| --- \| --- \| --- \| --- \| |
| Q21) Was the described population pharmacokinetics approach and validation method appropriate for the analysis?  Example:   - Population pharmacokinetics approach - Standard two-stage - Naive pooling of data - Mixed-effects modeling - Basic internal methods - Goodness-of-fit plots/diagnostic plots - Uncertainty in parameter estimates - Model sensitivity to outliers - Advanced internal methods - Data splitting - Bootstrap - Cross validation - Simulations such as visual or posterior predictive checks (PPCs) - External model evaluation (validation dataset observations compared with model predictions). | \| 1 \| 2 \| 3 \| 4 \| 5 \| \| --- \| --- \| --- \| --- \| --- \| |

| **Questions** | **5 Points Likert-scale** |
| --- | --- |
| Q22) Did the authors justify the selection of the key models at different stages of the development process?   - Justification of key models’ selection at different stages of the development process through using Goodness-of-fit (GOF) plots: - Predicted data versus observed data (PRED versus DV; a line of identity and a trendline should be included). - PRED versus weighted residuals (WRES; zero line and a trend line should be included). - Time versus WRES (a zero line and a trend line should be included). Time can be both time after dose and continuous time (time in the study). | \| 1 \| 2 \| 3 \| 4 \| 5 \| \| --- \| --- \| --- \| --- \| --- \| |
| Q23) Was the approval number provided by a regional Research Ethics Board stated? | \| 1 \| 2 \| 3 \| 4 \| 5 \| \| --- \| --- \| --- \| --- \| --- \| |
| Q24) The time duration from study submission to publication was less than 1 year. | \| 1 \| 2 \| 3 \| 4 \| 5 \| \| --- \| --- \| --- \| --- \| --- \| |
| Q25) Was a detailed description or reference of the specific level of statistical significance and the sample size calculations provided before the initiation of the study to ensure adequate power for detecting differences of  interest? | \| 1 \| 2 \| 3 \| 4 \| 5 \| \| --- \| --- \| --- \| --- \| --- \| |
| Q26) Were the chosen statistical tests and software to perform the statistical analysis appropriate to achieve the study objectives? | \| 1 \| 2 \| 3 \| 4 \| 5 \| \| --- \| --- \| --- \| --- \| --- \| |
| Q27) Was a patient flow diagram detailed to fully understand patient logistics?  Example:   - Number of patients who enrolled in each arm of the trial - Description of withdrawals | \| 1 \| 2 \| 3 \| 4 \| 5 \| \| --- \| --- \| --- \| --- \| --- \| |
| Q28) **Were the baseline characteristics of the included participants representative of the population of interest?**   - The following variables should be clearly defined for all participants’: sex, race, age, weight, height, concomitant diseases, co-medication, smoking habits, severity of illness, renal function, and hepatic function. | \| 1 \| 2 \| 3 \| 4 \| 5 \| \| --- \| --- \| --- \| --- \| --- \| |

| **Questions** | **5 Points Likert-scale** |
| --- | --- |
| Q29) In the event of missing data or outliers, was the process for analysis clearly justified? | \| 1 \| 2 \| 3 \| 4 \| 5 \| \| --- \| --- \| --- \| --- \| --- \| |
| Q30) Was an appropriate measure of precision (e.g. descriptive statistics confidence interval, standard deviation, mean, median, range, interquartile range, and trimmed range) used to document the pharmacokinetics results? | \| 1 \| 2 \| 3 \| 4 \| 5 \| \| --- \| --- \| --- \| --- \| --- \| |
| Q31) Were the study limitations described by the authors consistent with those identified within the study? | \| 1 \| 2 \| 3 \| 4 \| 5 \| \| --- \| --- \| --- \| --- \| --- \| |
| Q32) Were the author interpretations of the data consistent with the reported results? | \| 1 \| 2 \| 3 \| 4 \| 5 \| \| --- \| --- \| --- \| --- \| --- \| |
| Q33) Did the authors compare their observed results with the results of other relevant studies? | \| 1 \| 2 \| 3 \| 4 \| 5 \| \| --- \| --- \| --- \| --- \| --- \| |
| Q34) Were recommendations of future studies justified based on the results of this study? | \| 1 \| 2 \| 3 \| 4 \| 5 \| \| --- \| --- \| --- \| --- \| --- \| |
| Q35) Were the provided conclusions supported by the observed results?   - Authors should not provide any new information in the conclusion. | \| 1 \| 2 \| 3 \| 4 \| 5 \| \| --- \| --- \| --- \| --- \| --- \| |
| Q36) The authors referenced at least 10 other studies in order to defend their conclusions? | \| 1 \| 2 \| 3 \| 4 \| 5 \| \| --- \| --- \| --- \| --- \| --- \| |
| Q37) Were reported funding resources likely to influence the results of the study? | \| 1 \| 2 \| 3 \| 4 \| 5 \| \| --- \| --- \| --- \| --- \| --- \| |
| Q38) Were disclosed conflicts of interest likely to influence the results of the study? | \| 1 \| 2 \| 3 \| 4 \| 5 \| \| --- \| --- \| --- \| --- \| --- \| |
| Q39) In the final version of this proposed tool, which rating system do you feel would be the best method to help potential users appraise a clinical pharmacokinetics study? | \| 1 \| 2 \| 3 \| 4 \| 5 \| \| --- \| --- \| --- \| --- \| --- \| |

| **Questions** | **5 Points Likert-scale** |
| --- | --- |
| Q40) Do you think any other questions should be added to this appraisal tool that would help users appraise? | \| 1 \| 2 \| 3 \| 4 \| 5 \| \| --- \| --- \| --- \| --- \| --- \| |
| Demographics |  |
| 41) How many years of experience do you have in the field of clinical pharmacokinetics as a researcher or clinician? | \| 1 \| 2 \| 3 \| 4 \| 5 \| \| --- \| --- \| --- \| --- \| --- \| |
| 42) In your personal opinion, please rate your overall knowledge in the field of clinical pharmacokinetics? | \| 1 \| 2 \| 3 \| 4 \| 5 \| \| --- \| --- \| --- \| --- \| --- \| |
