# Supplementary material for: The use of a modified Delphi technique to develop a critical appraisal tool for clinical pharmacokinetic studies
Source: Int J Clin Pharm. 2022 Mar 20;44(4):894–903. doi: 10.1007/s11096-022-01390-y (PMC9393138; doi:10.1007/s11096-022-01390-y)
Supplement: Supplementary file 2 — Supplementary Material 2 [file 11096_2022_1390_MOESM2_ESM.docx]

| **Supplement 2:** Assessment of the interrater agreement per paper for clinical pharmacokinetics critical appraisal tool | | | | | | | | |
| --- | --- | --- | --- | --- | --- | --- | --- | --- |
| **Paper number** | **Name of the article** | **Rater-1** | **Rater -2** | **Time by rater-1** | **Time by rater-2** | **Kappa value** | ***p*-value** | **Percentage of agreement** |
| 1 | A randomized, placebo-controlled, single ascending-dose study to assess the safety, tolerability, pharmacokinetics, and immunogenicity of subcutaneous tralokinumab in Japanese healthy volunteers | SP | KJ | 16 min | 15 min | K 0.538 | 0.011* | 17/21*100 = 80.95% |
| 2 | Infliximab Pharmacokinetics are Influenced by Intravenous Immunoglobulin Administration in Patients with Kawasaki Disease | OR | KJ | 20 min | 15 min | K 0.176 | 0.375 | 13/21*100 = 61.90% |
| 3 | An Open-Label Crossover Study of the Pharmacokinetics of the 60-mg Edoxaban Tablet Crushed and Administered Either by a Nasogastric Tube or in Apple Puree in Healthy Adults | OR | KJ | 45 min | 10 min | K 0.859 | 0.000* | 20/21*100 = 95.24% |
| 4 | Identification of Cytochrome P450-Mediated Drug–Drug Interactions at Risk in Cases of Gene Polymorphisms by Using a Quantitative Prediction  Model | SP | KJ | 23 min | 10 min | K 0.897 | 0.000* | 20/21*100 = 95.24% |
| *significant *p*-value ≤ 0.05 | | | | | | | | |
| **Paper number** | **Name of the article** | **Rater-1** | **Rater -2** | **Time by rater-1** | **Time by rater-2** | **Kappa value** | ***p*-value** | **Percentage of agreement** |
| 5 | Pharmacokinetics, Safety and Tolerability of Oral Semaglutide in Subjects with Renal Impairment | OR | KJ | 30 min | 15 min | K 0.417 | 0.019* | 17/21*100 = 80.95% |
| 6 | Phase I Clinical Study of ZYAN1, A Novel Prolyl-Hydroxylase (PHD) Inhibitor to Evaluate the Safety, Tolerability, and Pharmacokinetics Following Oral Administration in Healthy Volunteers | OR | KJ | 90 min | 10 min | K 0.667 | 0.002* | 18/21*100 = 85.71% |
| 7 | Population pharmacokinetics and exposure–response modeling and simulation for evolocumab in healthy volunteers and patients with hypercholesterolemia | OR | KJ | 50 min | 15 min | K 0.386 | 0.075 | 16/21*100 = 76.19% |
| 8 | Population Pharmacokinetics Analysis of Alirocumab in  Healthy Volunteers or Hypercholesterolemic Subjects Using a Michaelis–Menten  Approximation of a Target-Mediated Drug Disposition Model—Support for a Biologics  License Application Submission: Part I | SP | KJ | 21 min | 15 min | K 0.152 | 0.368 | 13/21*100 = 61.90% |
| *significant *p*-value ≤ 0.05 | | | | | | | | |
| **Paper number** | **Name of the article** | **Rater-1** | **Rater -2** | **Time by rater-1** | **Time by rater-2** | **Kappa value** | ***p*-value** | **Percentage of agreement** |
| 9 | Pharmacokinetics of the B-Cell Lymphoma 2 (Bcl-2) Inhibitor Venetoclax in Female Subjects with Systemic Lupus Erythematosus | OR | KJ | 20 min | 15 min | K 0.829 | 0.000* | 20/21*100 = 95.24% |
| 10 | Population Pharmacokinetics of Volasertib Administered in Patients with Acute Myeloid Leukaemia as a Single Agent or in Combination with Cytarabine | OR | KJ | 45 min | 15 min | K 0.481 | 0.022* | 17/21*100 = 80.95% |
| 11 | A Population Pharmacokinetics and Pharmacodynamic Analysis of Abemaciclib in a Phase I Clinical Trial in Cancer Patients | OR | KJ | 60 min | 15 min | K 0.588 | 0.003* | 18/21*100 = 85.71% |
|  |  |  |  |  |  |  |  |  |
| 12 | Higher Midazolam Clearance in Obese Adolescents Compared with Morbidly Obese Adults | OR | KJ | 30 min | 15 min | K 0.632 | 0.004* | 18/21*100 = 85.71% |
| 13 | Pharmacokinetics Optimization of Everolimus Dosing in Oncology: A Randomized Crossover Trial | SP | KJ | 24 min | 15 min | K 0.475 | 0.030* | 17/21*100 = 80.95% |
| 14 | Population pharmacokinetics–pharmacodynamics of oral everolimus in patients with seizures associated with tuberous sclerosis complex | SP | KJ | 16 min | 15 min | K 0.152 | 0.475 | 13/21*100 = 61.90% |
| *significant *p*-value ≤ 0.05 | | | | | | | | |
| **Paper number** | **Name of the article** | **Rater-1** | **Rater -2** | **Time by rater-1** | **Time by rater-2** | **Kappa value** | ***p*-value** | **Percentage of agreement** |
| 15 | Population Pharmacokinetics and Optimal Sampling Strategy for Model-Based Precision Dosing of Melphalan in Patients Undergoing Hematopoietic Stem Cell Transplantation | OR | KJ | 75 min | 15 min | K 0.859 | 0.000* | 20/21*100 = 95.23% |
| 16 | Effects of Mild to Severe Hepatic Impairment on the  Pharmacokinetics of Sonidegib: A Multicenter, Open-Label, Parallel-Group Study | SP | AS | 45 min | 55 min | K 0.173 | 0.361 | 16/21*100 =76.19% |
| 17 | Pharmacokinetics of MHAA4549A, an Anti-Inﬂuenza A  Monoclonal Antibody, in Healthy Subjects Challenged with Inﬂuenza A Virus in a Phase IIa Randomized Trial | SP | AS | 18 min | 50 min | K 0.690 | 0.001* | 18/21*100 = 85.71% |
| 18 | Clinical Pharmacokinetics and Dose Recommendations  for Posaconazole in Infants and Children | SP | AS | 17 min | 40 min | K 0.640 | 0002* | 18/21*100 = 85.71% |
| 19 | Population Pharmacokinetics Modeling of  Olaratumab, an AntiPDGFRa Human Monoclonal Antibody, in Patients with Advanced and/or Metastatic Cancer | OR | AS | 45 min | 35 min | K 0.696 | 0.001* | 19/21*100 = 90.47% |
| *significant *p*-value ≤ 0.05 | | | | | | | | |
|  |  |  |  |  |  |  |  |  |
| **Paper number** | **Name of the article** | **Rater-1** | **Rater -2** | **Time by rater-1** | **Time by rater-2** | **Kappa value** | ***p*-value** | **Percentage of agreement** |
| 20 | Pharmacokinetics of dexmedetomidine during  analgosedation in ICU patients | SP | AS | 21 min | 30 min | K 0.323 | 0.129 | 18/21*100 = 85.71% |
| 21 | Pharmacokinetics of ADS-5102 (Amantadine) Extended Release Capsules Administered Once Daily at Bedtime for the Treatment of Dyskinesia | OR | AS | 30 min | 50 min | K 0.615 | 0.004* | 17/21*100 = 80.95% |
| 22 | Effect of Semaglutide on the Pharmacokinetics of  Metformin, Warfarin, Atorvastatin and Digoxin in Healthy Subjects | SP | AS | 18 min | 30 min | K 0.667 | 0.002* | 18/21*100 = 85.71% |
| 23 | Population Pharmacokinetics Modeling of JNJ-  53718678, a Novel Fusion Inhibitor for the Treatment of Respiratory Syncytial Virus: Results from a Phase I, Double-Blind, Randomized, Placebo-Controlled First-in-Human Study in Healthy Adult Subjects | SP | AS | 23 min | 30 min | K 0.314 | 0.115 | 16/21*100 = 76.19% |
| 24 | Characterization of the Pharmacokinetics of Vilaprisan:  Bioavailability, Excretion, Biotransformation, and Drug–Drug Interaction Potential | SP | AS | 16 min | 25 min | K 0.357 | 0.102 | 15/21*100= 71.43% |
| *significant *p*-value ≤ 0.05 | | | | | | | | |
| **Paper number** | **Name of the article** | **Rater-1** | **Rater -2** | **Time by rater-1** | **Time by rater-2** | **Kappa value** | ***p*-value** | **Percentage of agreement** |
| 25 | Piperacillin Population Pharmacokinetics and Dosing Regimen Optimization in Critically Ill Children with Normal and Augmented Renal Clearance | OR | AS | 30 min | 40 min | K 0.774 | 0.000* | 20/21*100=95.24% |
| 26 | Safety and Tolerability of Intravenous Valproic Acid in Healthy Subjects: A Phase I Dose-Escalation Trial | OR | AS | 60 min | 32 min | K 0.444 | 0.040* | 16/21*100 = 76.19% |
| 27 | Population Pharmacokinetics of GemtuzumabOzogamicin in Pediatric Patients with Relapsed or Refractory Acute Myeloid Leukemia | SP | AS | 27 min | 25 min | K 0.632 | 0.0004* | 18/21*100 = 85.71% |
| 28 | Clinical Pharmacokinetics and Mass Balance of Veliparib in Combination with Temozolomide in Subjects with Nonhematologic Malignancies | SP | AS | 22 min | 25 min | K 0.222 | 0.292 | 15/21*100 = 71.43% |
| 29 | Model-Based Therapeutic Drug Monitoring of Inﬂiximab  Using a Single Serum Trough Concentration | SP | AS | 26 min | 23 min | K 0.533 | 0.015 | 17/21*100 = 80.95% |
| 30 | The Ontogeny of UDP-glucuronosyltransferase Enzymes, Recommendations for Future Profiling Studies and Application Through Physiologically Based Pharmacokinetics Modelling | OR | AS | 30 min | 17 min | K 0.774 | 0.000* | 20/21*100= 95.24% |
