# Supplementary material for: The use of a modified Delphi technique to develop a critical appraisal tool for clinical pharmacokinetic studies
Source: Int J Clin Pharm. 2022 Mar 20;44(4):894–903. doi: 10.1007/s11096-022-01390-y (PMC9393138; doi:10.1007/s11096-022-01390-y)
Supplement: Supplementary file 3 — Supplementary Material 3 [file 11096_2022_1390_MOESM3_ESM.docx]

# CACPK Tool: Critical Appraisal of Clinical Pharmacokinetic Studies

**Evaluator’s name: _________________________________________________**

**Name of the evaluated article: ________________________________________**

**Time to complete evaluation of article: _________________________________**

**Number of YES responses: ___________________________________________**

| **CACPK Tool: Critical Appraisal of Clinical Pharmacokinetic Studies** | |
| --- | --- |
| ***Appraising Background*** | |
| 1. Was a clear description of the objectives of the study provided?  - Authors should provide a clear statement of the objectives of the research to clarify the purpose and the scope of the study. | Yes  No  I Do Not Know  Not Applicable  Comments: ______________________________ |
| 1. Was a clear and comprehensive rationale provided to support the purpose of the study? | Yes  No  I Do Not Know  Not Applicable  Comments: ______________________________ |
| ***Appraising Study Design and Experimental Methods*** | |
| 1. Was the chosen study design appropriately selected and justified? | Yes  No  I Do Not Know  Not Applicable  Comments: ______________________________ |
| 1. Was the dosing (i.e. dose, route of administration, and dosing interval) of the drug in the study justified for the intended study?   **Examples:**   - Authors should justify the use of single-dose versus steady-state analysis. | Yes  No  I Do Not Know  Not Applicable  Comments: ______________________________ |
| 1. Were the outcome measures endpoints of the study appropriate to address the objectives of the study? | Yes  No  I Do Not Know  Not Applicable  Comments: ______________________________ |
| 1. Were the exclusion criteria of participants included AND appropriate for the intended outcomes of the study?  - The exclusion criteria should be relevant to assist with decreasing significant confounders (e.g. co-administration of drugs, organ impairment, and special populations) that may impact outcomes. | Yes  No  I Do Not Know  Not Applicable  Comments: ______________________________ |
| 1. Where applicable, were the relevant baseline characteristics of the participants adequately described?   **Examples:**   - Sex, race, age, weight, height, concomitant disease, administered medications, smoking status, pregnancy, severity of illness that may affect pharmacokinetic parameters, renal function, and hepatic function.   **Note:** Please refer to Appendix-1 Patient Demographics for further clarification. | Yes  No  I Do Not Know  Not Applicable  Comments: ______________________________ |
| 1. Were plausible interacting covariates described *a priori* or in post hoc evaluation?   **Examples:**   - Demographic variables, laboratory values, concomitant medications, and relevant disease states to the drug being studied. | Yes  No  I Do Not Know  Not Applicable  Comments: ______________________________ |
| 1. Was the description of the used biological sample analytical methods sample analysis methods or citations of prior validation studies provided in the publication or affiliated appendix?   **Examples:**   - Chromatography type. - Detection type. - Assay characteristics: mobile phase composition, gradient and flow rate, chromatographic column (packing material, dimensions). - Analytical runtime. - Operating temperature. - Detection parameters. - Validation method: specificity, recovery, linearity and sensitivity, the stability of the assay and its reproducibility. Refer also to EMA/FDA guidelines for bioanalytical method validation. | Yes  No  I Do Not Know  Not Applicable  Comments: ______________________________ |
| 1. Was the method of data sampling of analytics appropriate for the study?   **Examples:**   - First vs. second order absorption, and lag time. - Evaluating for nonlinearity requires multiple dose levels and a complete profile is recommended. - Researchers obtain these data from previously conducted studies with completed concentration-time profile (e.g. phase I studies). - The method of data sampling should reference previously validated quantitative bioanalytical methods and if those are not available then the full description or defense of data sampling should be included. | Yes  No  I Do Not Know  Not Applicable  Comments: ______________________________ |
| 1. Was a clear description of the sampling site provided and justified?   **Examples:**   - Sampling site should be consistent for all subjects in the study. - Arterial sampling is preferable during frequent sampling schedule. - Arterial sampling is more representative of the delivered concentration to the effect site in the case of peripheral elimination. - Arterial sampling is preferable when administering a drug that has a short duration of action or fast onset of action. | Yes  No  I Do Not Know  Not Applicable  Comments: ______________________________ |
| 1. Was the number of half-lives elapsed within the sampling period appropriate for the analyzed drug?   **Examples:**   - Sampling interval should not exceed the expected half-life of the studied exponential phase (fast distribution, slow distribution and elimination). | Yes  No  I Do Not Know  Not Applicable  Comments: ______________________________ |
| 1. Were sample storage conditions appropriate and described in a manner that could be accurately replicated?   **Examples:**   - Sample storage, temperature, use and description of anticoagulants, stabilizers, centrifugation etc. | Yes  No  I Do Not Know  Not Applicable  Comments: ______________________________ |
| 1. If applicable, was there a clear description of the pharmacokinetic model, its development, validation and justification for use?   It is recommended to provide the following details about the selected modeling process:   - Description of studies from which dataset was driven - Model structure - Validated software for the pharmacokinetic analysis - Criteria for accepting valid model’s parameters - Fitting procedure defined prior to the initiation of the analysis. - A reasonable assumption based on which the scheme for weighting is considered to be appropriate and the transformation of data [e.g. logarithmic transformation to achieve the homoscedastic (constant) variance requirements] should be provided. | Yes  No  I Do Not Know  Not Applicable  Comments: ______________________________ |
| 1. Was the described population pharmacokinetic approach validation method appropriate for the analysis? 2. Basic internal method 3. Advanced internal method 4. External model evaluation   **Note**: Please refer to Appendix-2 Model Evaluation for further clarification. | Yes  No  I Do Not Know  Not Applicable  Comments: ______________________________ |
| 1. Were the essential pharmacokinetic parameters required to make the results applicable in clinical settings included?   **Examples:**   - Total clearance (CL), Hepatic clearance, Renal clearance, Volume of distribution at steady state (Vss), Blood/plasma concentration ratio, Terminal half-life (t_1/2_ Z), Fraction of the unbound drug in plasma (fu), Absorption rate constant (Ka),C_min_, C_max_, t_max_, , AUC, etc. | Yes  No  I Do Not Know  Not Applicable  Comments: ______________________________ |
| 1. Were the pharmacokinetic equations used to calculate the patient’s pharmacokinetic parameters presented or cited within the article?   **Examples:**   - Equations used to calculate the following pharmacokinetic parameters: creatinine clearance, body weight calculations, Michaelis Menten, volume of distribution | Yes  No  I Do Not Know  Not Applicable  Comments: ______________________________ |
| ***Appraising Applied Statistics*** | |
| 1. Were the chosen statistical tests and software to perform the statistical analysis appropriate to achieve the study objectives? | Yes  No  I Do Not Know  Not Applicable  Comments: ______________________________ |
| ***Appraising Results*** | |
| 1. Were all patients enrolled in the study accounted for?   **Examples:**   - Description of patient screening, enrollment, run-in or wash out phases, study period and follow-up periods are adequately described. Any loss to follow-up or withdrawals are described. | Yes  No  I Do Not Know  Not Applicable  Comments: ______________________________ |
| 1. In the event of missing data or outliers, was the process for analysis justified and appropriate? | Yes  No  I Do Not Know  Not Applicable  Comments: ______________________________ |
| 1. Were appropriate summary statistics to describe centrality and variance used to present the pharmacokinetic results?   **Examples:**   - Descriptive statistics such as confidence interval, standard deviation, mean, median, range, interquartile range, standard error and trimmed range | Yes  No  I Do Not Know  Not Applicable  Comments: ______________________________ |
