# Supplementary material for: The use of a modified Delphi technique to develop a critical appraisal tool for clinical pharmacokinetic studies
Source: Int J Clin Pharm. 2022 Mar 20;44(4):894–903. doi: 10.1007/s11096-022-01390-y (PMC9393138; doi:10.1007/s11096-022-01390-y)
Supplement: Supplementary file 4 — Supplementary Material 4 [file 11096_2022_1390_MOESM4_ESM.docx]

# Appendix-1 in the CACPK tool

This appendix has been added based on comments. It is not intended as a question to be answered by users of this tool, but more as a reference should one be required. Please rate your agreement with having this appendix available to users of this tool.

All the following variables should be clearly defined for all participants: sex, race, age, weight, height, concomitant diseases, co-medication, smoking habits, covariates, the severity of illness, residual, renal function, and hepatic function. Authors should describe if participants are taking any medications that may interact with the analyzed medication.

The Acute Dialysis Quality Initiative (ADQI) minimum reporting criteria by ADQI should be followed in case of including participants on dialysis.

a) Operational characteristics

- Membrane/ dialyzer/filter and area
- A measure of time spent on therapy
- Delivery device
- Access and blood flow
- Anticoagulation
- Replacement fluid composition and administration
- Dialysis fluid composition and administration

b) Patient characteristics

- A measure of time spent on therapy
- Surgical/trauma/medical/other
- A measure of the severity of illness
- Cointerventions
- Integrated hemodynamic status and vasopressor treatment
- Outcomes

In the case of including participants suffering from renal failure the following information should be provided:

- Cause
- Plasma creatinine concentration/creatinine clearance
- Plasma electrolytes
- Hemoglobin concentration
- Plasma protein level
- Time and the nature of last dialysis
- Existence of clinical edema
- Existence of peripheral neuropathy

In case of including participants who have hepatic cirrhosis the following information should be provided:

- Cause
- Child’s Pugh Score
- Prothrombin time, platelet count
- Albumin and globulin levels

In case of including critically ill participants following information should be provided:

- Clinical description
- Apache II score
- Plasma creatinine and electrolyte concentrations
- Presence/absence of renal failure
- Presence/absence of liver failure

In case of including participants suffering from thermal injury the following information should be provided:

- Regular hematocrit
- Preoperative plasma albumin and globulin level

In the case of Bioequivalence studies, the following criteria should be fulfilled:

- Nonsmoker healthy volunteers (males/females) with a body weight that is ± 20% of the standard and with age between 18 to 55 years old should be enrolled.
