# Supplementary material for: The use of a modified Delphi technique to develop a critical appraisal tool for clinical pharmacokinetic studies
Source: Int J Clin Pharm. 2022 Mar 20;44(4):894–903. doi: 10.1007/s11096-022-01390-y (PMC9393138; doi:10.1007/s11096-022-01390-y)
Supplement: Supplementary file 5 — Supplementary Material 5 [file 11096_2022_1390_MOESM5_ESM.docx]

# Appendix-2 in the CACPK tool

| **Model evaluation / validation and diagnostics** | **Methods** | **Description of use** |
| --- | --- | --- |
| **Basic internal methods** | | |
| Goodness-of-fit plots/diagnostic: assessments of the goodness of fit statistics/ plots | The following predicted data should be plotted versus the observed data: PRED (population prediction) vs. DV (dependent variable), PRED vs. WRES (weighted residuals) or CWRES (conditional weighted residuals), and time vs. WRES or CWRES | Different models were selected based on the observed goodness-of-fit in the diagnostic plots. |
| Internal validation | Data splitting: randomly the data are separated into an index population and a test population | Experts use data splitting to determine if the used model was robust. |
| Model reliability: to assess parameters uncertainty and its random effect; the plausibility of parameter estimates and their precision  Model stability: to identify the extent to which the model is resistant to change | Log-likelihood profiling: mapping the objective function; considered as another method for determining parameter CI | Use the likelihood profiling to enhance the model fit through models evaluation through considering the change in both the objective function and determination of the empirical 95% CI. |
| Resampling techniques | Bootstrapping technique is used to estimate 95% CIs and standard error of the estimate. Bootstrap generates other plausible data and assesses model structure.  Jack-Knife techniques: estimates the standard error of estimates.  Cross-validation | Used for the validation of the final model.  Cross-validation is done through using Jack-Knife techniques to assess the accuracy and the validity of the pharmacokinetic final model.  Cross-validation is used to determine the robustness and ability of the final model to predict data. Randomly, a full dataset was divided |
| Case-deletion diagnostics | Determine and assess the important outliers’ effect | Case-deletion diagnostics are used to evaluate the final model by detecting influential individual and assessing its robustness. |
| **Advanced internal method** | | |
| Simulation-based diagnostics | VPC (visual predictive check): this is a plot used to compare the 95% prediction interval to the observed data  NPC (Numerical predictive check): this is used to assess the appropriateness of the model  NPDE (Normalized prediction distribution error)  PPC (posterior predictive check): this is used to assess and determine the predictive model performance | VPC: used to determine the accuracy and the performance of the used model for data description.  NPC: used to assess the final model simulation properties.  NPDE: used to produce 1000 model-prediction concentration for each observation available in the external dataset. After that, the observed concentration compared to the 1000 predicted concentrations.  PPC: used to determine if the used model helps in extensively describing both the covariate disposition and the pharmacokinetic parameters. |
| **External model validation** | | |
| External validation | A validation dataset from another study used to test the developed model | The external validation process assesses the developed final model. |
